# Video Game Addiction in Gambling Disorder: Clinical, Psychopathological, and Personality Correlates

**DOI:** 10.1155/2014/315062

**Published:** 2014-07-14

**Authors:** Susana Jiménez-Murcia, Fernando Fernández-Aranda, Roser Granero, Mariano Chóliz, Melania La Verde, Eugenio Aguglia, Maria S. Signorelli, Gustavo M. Sá, Neus Aymamí, Mónica Gómez-Peña, Amparo del Pino-Gutiérrez, Laura Moragas, Ana B. Fagundo, Sarah Sauchelli, José A. Fernández-Formoso, José M. Menchón

**Affiliations:** ^1^Department of Psychiatry, University Hospital of Bellvitge-IDIBELL, 08907 Barcelona, Spain; ^2^Ciber Fisiopatologia Obesidad y Nutrición (CIBERobn), Instituto Salud Carlos III, 08907 Barcelona, Spain; ^3^Department of Clinical Sciences, School of Medicine, University of Barcelona, 08907 Barcelona, Spain; ^4^Department of Psychobiology and Methodology of Health Science, Universitat Autònoma de Barcelona, 08193 Barcelona, Spain; ^5^School of Psychology, University of Valencia, 46010 Valencia, Spain; ^6^Department of Clinical and Molecular Biomedicine, Institute of Psychiatry, University of Catania, 95100 Catania, Italy; ^7^Institute of Sociology, University of Porto, 4150-564 Porto, Portugal; ^8^Departament of Nursing Public Health, School of Nursing, University of Barcelona, 08907 Barcelona, Spain; ^9^CIBER, Spanish Institute of Health Carlos III (ISCIII), 28029 Madrid, Spain; ^10^CIBER Salud Mental (CIBERSAM), Instituto de Salud Carlos III, 28029 Madrid, Spain

## Abstract

*Objective*. We studied the prevalences of video game use (VGU) and addiction (VGA) in gambling disorder (GD) patients and compared them with subjects with non-video game use (non-VGU) in relation to their gambling behavior, psychopathology, and personality characteristics. *Method*. A sample of 193 GD patients (121 non-VGU, 43 VGU, and 29 VGA) consecutively admitted to our pathological gambling unit participated in the study. *Assessment*. Measures included the video game dependency test (VDT), symptom checklist-90-revised, and the temperament and character inventory-revised, as well as a number of other GD indices. *Results*. In GD, the observed prevalence of VG (use or addiction) was 37.3% (95% CI :30.7% ÷ 44.3),VGU 22.3% (95% CI :17.0% ÷ 28.7), and VGA 15% (95% CI :10.7% ÷ 20.7). Orthogonal polynomial contrast into logistic regression showed positive linear trends for VG level and GD severity and other measures of general psychopathology. After structural equation modeling, higher VG total scores were associated with younger age, general psychopathology, and specific personality traits, but not with GD severity. Patients' sex and age were involved in the mediational pathways between personality traits and VG impairment. *Conclusions*. GD patients with VG are younger and present more dysfunctional personality traits, and more general psychopathology. The presence of VG did not affect the severity of GD.

## 1. Introduction

Research on gambling disorder (GD) is relatively new. In fact it was not until 1980 that the diagnostic and statistical manual of mental disorders, in its third edition (DSM-III), formally recognized this disorder (then was called pathological gambling) and included it in impulse- control disorders not elsewhere classified. Recently, in the DSM-5 [1], the nosological nature of the disorder was changed after reviewing the existing literature and evidence [2]; it was renamed as gambling disorder (GD) and classified in a new section called* Substance Related and Addictive Disorders*. Moreover, the illegal acts criterion was removed, the cut-off for the diagnosis of GD was modified from five to four criteria, and it was specified that symptoms had to be present for a period of 12 months [3].

During the review of the manual all possible nonsubstance addictions were analyzed, that is, pathological gambling, internet gaming, more general use of the Internet, shopping, exercise, and work. Finally, only GD was classed as a nonsubstance addiction, due to its clinical similarities, phenomenology, comorbidity, and treatment response with substance use disorders (SUDs) and also due to its shared neurobiological factors [4, 5].

However, the working committee of the DSM-5 decided to place Internet gaming disorder (IGD) in Section 3, which includes potential problems that require further investigation. This decision was based on the growing number of clinical and population studies of the disorder and its severe individual and interpersonal consequences [6]. Additionally, certain similarities in neurobiological features [7, 8], psychiatric comorbidity, and personality traits (sensation seeking, impulsivity, and low self-esteem) have recently been found between IGD with SUDs and GD [9]. Given that a wide range of tools and criteria have been used in the IGD scientific literature, it was decided to establish a set of nine diagnostic criteria, of which five or more must be present for a period of 12 months in order to standardize the definition and diagnosis of IGD [2, 6]. The inclusion of this condition in the DSM-5 will undoubtedly have a significant impact not only on future research [10] but also on the more clinical aspects such as destigmatization and improvements in diagnosis and treatment [11].

Although game users in industrialized countries tend to be over 18 [12], few studies have explored IGD in adult populations. Most of the ones carried out to date have been conducted in Europe [13–16]. All coincide in indicating the association between the use of massively multiplayer online role-playing games (MMORPGs) and problematic or addictive behavior. Prevalence rates range between 0.2% and 1.3% for addictive use and 3.3% and 4.1% for problematic behavior [14–16]. However, the study by Achab et al. [13] in an adult population, which adapted the DSM-IV-TR diagnostic criteria [17] for substance dependence disorders to MMORPGs, reported an addiction rate as high as 27.5%. The disparity of the results may be due to the differences in the assessment tools used by the studies or in the target population investigated (as suggested by King et al. [18]); while some studies concentrated on specific adult users more prone to developing addictive behaviors [13], others concentrated on young populations [19, 20]. However, several authors noted specific factors common to all participants (e.g., withdrawal, loss of control, high rates of tolerance, social and financial problems, problems with relatives, as well as mood swings, anxiety, irritability, sedentary lifestyle, decreased sleep, and abandonment of obligations, responsibilities, and leisure activities) [6, 11, 16, 18].

Other sociodemographic and clinical variables associated with adult IGD were age (the condition being more common in younger adults), higher education, residence in urban areas, and early age of onset [13]. The same features have been described in GD [21, 22]. In addition, both disorders have been associated with psychopathology such as depression, anxiety, and impulse-control disorders [6, 11, 23] and with dysfunctional personality traits such as high impulsivity and sensation seeking, neuroticism, introversion, and hostility [11, 24, 25].

The few studies that have compared GD with general new technology addiction [26–29] coincide in reporting high levels of psychopathology and maladaptive personality traits in both disorders. However, most of them do not differentiate between IGD and the problem of more general use of the network or Internet addiction (IA). Tonioni et al. [28] reported not only similarities in relation to the association of depression, anxiety, and overall functioning but also differences in social patterns. Social skills were lower in the IA group, who presented lower social acceptance, cooperation, and social support in general. Regarding personality traits, both groups had low scores on reward dependence and self-directedness and high scores on self-transcendence. However, Muller et al. [29] identified higher neuroticism, lower conscientiousness, and extraversion in patients with IGD, the last two being statistical predictors of the condition. For Kuss [11], despite the existence of vulnerability factors common to the two disorders such as the involvement of brain reward circuits, impulsivity, deficits in executive functions, and attention, there were also marked clinical differences, apart from the preoccupation and obsessive use observed in both.

Although some studies have explored differences and commonalities between GD and IGD/VG, few have analyzed the use and abuse of VG in GD. Based on the results of previous studies [28], we hypothesized that there would be more similarities than differences between three groups of GD patients divided according to level of video game use: non-video game users (non-VGU), video game users (VGU), and video game addicts (VGA). However, we expected the group with GD plus VGA to display more severe psychopathology and dysfunctional personality traits (viz., higher levels of persistence, defined as perseverance in behavior despite frustration or fatigue).

Given the current lack of studies in clinical samples, especially in adult populations, the present study had three main goals: (1) to assess the current presence of video game addiction (VGA) symptoms in GD, (2) to establish whether the presence of VGA symptoms is associated with greater severity of GD symptomatology and general psychopathology, and (3) to assess whether the presence of more VGA symptoms is associated with specific temperament and character personality traits in GD patients.

## 2. Method

### 2.1. Participants

A total of 193 treatment-seeking GD patients participated in the current study (167 males and 26 females), consecutive referrals for assessment, and outpatient treatment at the Pathological Gambling Unit of the Psychiatric Department at the University Hospital of Bellvitge, Barcelona, Spain, 2013. All patients were diagnosed according to DSM-IV criteria using Stinchfield's diagnostic questionnaire for pathological gambling [30, 31], conducted by experienced psychologists and psychiatrists. The majority of GD patients were slot machine gamblers (63.7%; *N* = 123). According to the video game dependency test (VDT), GD patients were assigned post hoc to three groups: 121 (62.7%) with total VDT scores of 0 to the non-video game user group (non-VGU), 43 (22.3%) with total VDT scores between 1 and 19 to the video game user group (VGU), and 29 (15%) with total VDT scores 20 or more to the video game addict group (VGA). All were Internet gaming players.

As shown in Table 1, the mean age of the sample was 42.4 years old (SD = 13.4). Most subjects were employed (51.3%) and 33.2% were single or without a partner. Problem alcohol use was recorded in 18.1%, and substance abuse in 7.3%.

### 2.2. Instruments

A comprehensive assessment battery was administered which measured GD and VGA symptoms, sociodemographic characteristics, general psychopathology, and personality traits. The battery included internationally applied instruments in the GD field, such as the South Oaks Gambling Screen (SOGS) [32, 33] and Stinchfield's diagnostic questionnaire for pathological gambling according to DSM-IV criteria [30, 31]. A validated Spanish-language scale entitled video game dependency test (*Test de Dependencia de Videojuegos*—VDT) [34], the symptom checklist-revised (SCL-90-R) [35], and the temperament and character inventory-revised [36] were also used.

#### 2.2.1. South Oaks Gambling Screen (SOGS) [33]

The SOGS includes 20 items that produce a total score ranging from 0 to 20, with higher values indicating more severe psychopathology, and a score of five or more indicating probable pathological gambling (PG—now renamed as “gambling disorder” in DSM-5 [3, 37]). The psychometric properties of the Spanish version of the questionnaire have been shown to be satisfactory. Test-retest reliability was *r* = 0.98 and internal consistency was 0.94 (Cronbach's *α*). Convergent validity with regard to DSM-III-R criteria for pathological gambling [38] has been estimated at *r* = 0.92 [39]. Furthermore, several studies in both clinical and general population samples have reported that the SOGS presents satisfactory psychometric properties as an index of gambling problem severity [40–42].

#### 2.2.2. Stinchfield's Diagnostic Questionnaire for Pathological Gambling according to DSM-IV Criteria [30, 31]

This questionnaire measures the ten DSM-IV diagnostic criteria for PG with 19 items [43]. This scale has demonstrated satisfactory psychometric properties. Internal consistency, measured with Cronbach's alpha, yielded values of *α* = 0.81 for the general population and *α* = 0.77 for a gambling treatment group. Convergent validity was estimated with a correlation with the SOGS as *r* = 0.77 for a general population sample and *r* = 0.75 for a gambling treatment sample. This scale has been adapted for the Spanish population by Jimenez-Murcia, Stinchfield, and colleagues [31] and has demonstrated adequate psychometric properties. Cronbach's alpha in the present sample was very good (*α* = 0.90).

*Video game dependency test *(Test de Dependencia de Videojuegos—VDT) [34] is a reliable and valid 25-item self-report scale that assesses video game dependence and video game addiction. The test incorporates four factors that make up the principal characteristics of dependence: withdrawal, tolerance, problems caused by excessive use, and lack of control. Of these factors, as expected, withdrawal (defined as the distress arising from not being able to play video games and using games as a means of coping with adverse emotional states) accounts for the greatest part of the variance. The VDT total score is an indicator of video game addiction, with a cut-off score of 20. Internal consistency for the VG total score in the sample was excellent (alpha = 0.97). ROC procedures selected 20 as the best cut-off for the raw score, with a sensitivity of 80.0% and a specificity of 86.7% (area under the ROC curve = 0.80, *P* = 0.024).

#### 2.2.3. Temperament and Character Inventory-Revised (TCI-R) [36]

This is a 240-item questionnaire with 5-point Likert response options [44]. It measures seven dimensions of personality: four temperaments (harm avoidance, novelty seeking, reward dependence, and persistence) and three characters (self-directedness, cooperativeness, and self-transcendence). The Spanish version of the inventory has demonstrated satisfactory psychometric properties, ranging between 0.77 and 0.84 [45, 46].

#### 2.2.4. Symptom Check List 90-Item-Revised (SCL-90-R) [35]

The SCL-90-R measures a broad range of psychological problems and psychopathology symptoms. The questionnaire contains 90 items and measures nine primary symptom dimensions: somatization, obsessive/compulsive, interpersonal sensitivity, depression, anxiety, hostility, phobic anxiety, paranoid ideation, and psychoticism. It also includes three global indices: a global severity index (GSI), designed to measure overall psychological distress; a positive symptom distress index (PSDI), designed to assess symptom intensity; and a positive symptom total (PST), which reflects self-reported symptoms. The GSI can be used as a summary of the subscales. Evaluation of the revised Spanish-language version generated an internal consistency (coefficient alpha) of 0.75 [35, 47].

Additional demographic, clinical, and social/family variables related to gambling were evaluated using a semistructured face-to-face clinical interview described elsewhere [48].

### 2.3. Procedure

In accordance with our unit's assessment protocol and treatment model published elsewhere [48], we carried out a specific semistructured interview and functional analysis of GD. All the information was collected during the first interview. The remaining psychometric assessments mentioned above were administered to all subjects in a second session. Both interviews were conducted in a time frame of one week by a psychologist and a psychiatrist (each with more than 15 years of work experience in this field). GD patients were assigned to the three VG groups (non-VGU, VGU, and VGA) as described in Section 2.1 above. The Ethics Committee of the University Hospital of Bellvitge (Barcelona, Spain) approved the study, and informed consent was obtained from all participants.

### 2.4. Statistical Analysis

Analyses were carried out with SPSS20 for Windows. The three VG groups were compared through logistic regression for dichotomous outcomes and with ANOVA procedures for quantitative data. For both models (logistic regression and ANOVA), the VG groups were entered as independent variables and the variables measuring the GD related measures were considered the criteria. Orthogonal polynomial contrasts (used for grouping-ordered independent factors) performed a trend analysis to test patterns in data, the presence of linear and/or quadratic trends (*k* − 1 = 2 order comparisons were assessed, linear and quadratic trends, due to the *k* = 3 levels of the grouping variable). Cohen's *d* was used to measure the effect size for pairwise comparison between groups (effect size was considered low with |*d*| < 0.50, moderate with |*d*| > 0.50, and high with |*d*| > 0.80).

Partial correlations, adjusted for the participants' sex and age, evaluated the association between VG total score (considered as a dimensional-metric variable) and clinical measures.

Stepwise multiple regression and binary logistic regression selected the best predictors of the VG scores (for each scale and for the binary classification based on the cut-off = 20), considering as input variables the participants' sex, age, employment status, marital status, and personality profile (TCI-R scores).

The mediational hypotheses were tested through structural equation models (SEM) with STATA13 for Windows. Overall goodness-of-fit statistics were assessed through *χ* ^2^ test, the root mean squared error of approximation (RMSEA), baseline comparison index (comparative fit index CFI), and residual size (standardized mean squared residual SMSR). A fit was considered to be good if [49] a nonsignificant result (*P* > 0.05) was achieved in the *χ* ^2^ test, if the RMSEA was lower than .08, if the CFI coefficients were higher than 0.90, and if SRMR was limited to 0.08. The equation level goodness-of-fit and the effect sizes were also estimated through *R* ^2^ coefficients for each equation and for the global model (these coefficients evaluated the fraction of variance explained by the indicator/indicators), multiple correlation (mc), and Bentler-Raykov multiple correlation (mc^2^) [50]. These last two coefficients reflect the relatedness of each dependent variable with the model's linear prediction (in nonrecursive models, mc^2^ is computed to avoid the problem of obtaining inconsistent negative multiple correlations).

## 3. Results

### 3.1. Sociodemographic and Clinical Variables and Prevalence of VG

There were 121 non-VGU participants (62.7%, 95%CI: 55.7%–69.2%), 43 video game users (VGU) (22.3%, 95%CI: 17.0%–28.7%), and 29 video game addicts (VGA) (15.0%, 95%CI: 10.7%–20.7%). Table 1 includes the descriptive data of the total sample and the separate groups based on the video game questionnaire total raw scores. Statistical differences emerged for patients' age (with non-VGU patients being older) and the age of onset of the GD problem (with non-VGU patients also presenting older ages of onset).

There was insufficient evidence to conclude that mean VDT total scores differed according to participants' sex, employment status, marital status, use of tobacco, and use of substances.

### 3.2. Comparison between VG Groups for the GD Measures: SOGS and DSM-IV Questionnaires

The upper part of Table 2 shows the comparison of the SOGS scores (for each item and for the total score) between VG groups. The prevalence of patients who reported playing slot machines and other betting games was higher in the VGA group (*P* = 0.045 and *P* = 0.022). A positive linear trend was found for “playing cards” (the higher the VG level, the higher the prevalence of patients reporting this form of gambling) and a quadratic trend for the prevalence of other forms of betting (prevalences were 15.4, 5.3, and 31.8 for non-VGU, VGU, and VGA, resp.). The mean SOGS-total score presented a positive linear trend with the VG level (this means that it increased from 9.7 for non-VGU to 10.1 to VGU and 11.2 to VGA, *P* = 0.043).

According to the DSM-IV questionnaire results (lower part of Table 2), the VGA had a statistically higher prevalence of patients reporting the presence of criterion A2 (“needs to bet more money,” *P* = 0.002), and linear and quadratic trends were found for this symptom. A positive linear trend was found for criterion A6 (“gambles again after losing,” *P* = 0.050) and for the means for the DSM-total criteria (*P* = 0.038).

Effect size measured through Cohen's *d* showed that for the dichotomous SOGS-items and DSM-criteria the highest differences were between non-VGU and VGA patients (within the moderate range for significant group comparisons, except for the item “other forms of gambling” and the criterion “needs to gamble more money”) and the lowest between VGU and VGA patients. Differences between non-VGA and VGA achieved moderate effect sizes for the SOGS-total score and the DSM-total criteria, and the other pairwise comparison achieved a low effect size.

### 3.3. Comparison between VG Groups for General Psychopathology and Personality

Table 3 shows the results of the ANOVA procedures comparing the SCL-90-R and the TCI-R mean scores between the three VG groups. All the SCL-90-R scales achieved significantly different means between the three groups. The significant linear trends obtained in the polynomial contrasts indicated that the higher the VG scores, the higher the SCL-90-R mean score (VGA > VGU > non-VGU). The additional significant quadratic trend indicated that while the mean differences between non-VGU and VGU were low, the differences between VGU and VGA were high. Cohen's *d* measuring the effect size for pairwise SCL-90-R and TCI-R comparisons showed that differences between non-VGU and VGU were low (except for TCI-R persistence score). Pairwise differences for the rest of the SCL-90-R scales obtained moderate to high effect sizes. For TCI-R scores, moderate differences were obtained for the self-directedness score for the pairwise comparison between VGA patients and the other two VG levels.

A positive linear trend was also obtained for the relationship between the VG groups and the TCI-R mean score for persistence and a negative linear trend between the VG groups and the TCI-R mean scores for self-directedness. An additional quadratic trend for TCI-R self-directedness again showed low mean differences between non-VGU and VGU and higher mean differences between VGU and VGA.

### 3.4. Association between VG Scores and Clinical Outcomes

Partial correlations adjusted for the covariates patients' sex and age showed that VG total scores correlated positively with all the SCL-90-R scores and negatively with the TCI-R self-directedness score (Table 4). The effect sizes of the correlations were in the moderate range.

### 3.5. Predictive Capacity of the Sociodemographic and the Personality Traits among VG Groups

The first stepwise linear regression included in Table 5 contains the best predictive model selected for the VG total score, considering the sociodemographic variables and the personality profile measured via the TCI-R questionnaire as independent variables. The only significant predictor was the TCI-R self-directedness score: the lower the TCI-R self-directedness score was, the higher the VG total score was.

The second model in Table 5 corresponds to the stepwise binary logistic regression evaluating the best predictors (entering in the model the same set of independent variables as in the previous multiple regression) of a score higher than 0 on the VG total scale (the dependent variable was coded 0 for non-VGU patients and 1 for VGU and VGA patients). Results showed that a greater likelihood of a VG above 0 (VGU and VGA) was associated with younger age and high TCI-R persistence scores.

The third model in Table 5 contains the best model for discriminating a VG total score above 20 (the dependent variable was coded 0 for non-VGU and VGU patients and 1 for VGA patients). The results showed that low TCI-R self-directedness scores increased the risk of VGA.

### 3.6. Pathways of the VG Level and GD Behavior

**Figure 1:**
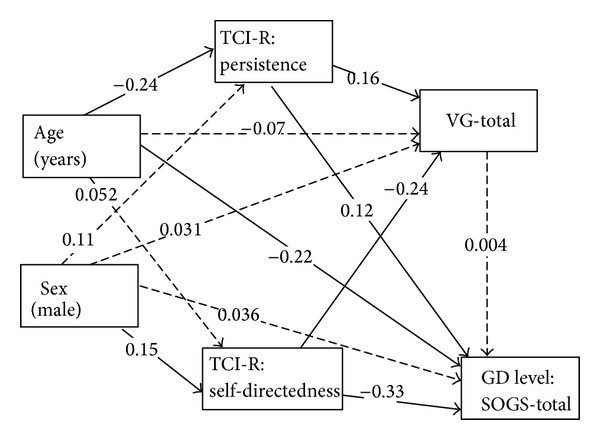
Structural equation model (SEM) valuing the pathways for the video game (VG) and the gambling disorder (GD) levels. Dashed lines indicate nonsignificant associations.

Figure 1 shows the diagram for the SEM that assesses the pathways for the outcomes VG behavior severity (measured through the VG total score) and severity of the GD (SOGS total score).  Table 6 includes the statistics for the standardized coefficients of this model. The variables included in the SEM were selected from the results obtained in the previous stepwise regression models, which identified patients' age and TCI-R persistence and self-directedness scores as the most relevant predictors for VG (sex was also included as an independent variable due to its strong association with GD). The dashed lines indicate nonsignificant links. The variables selected to adjust the pathway were the ones with the highest associations in the previous analyses. The indexes measuring the model level goodness-of-fit were adequate: *χ* ^2^ = 0.29 (*P* = 0.589), RMSEA = 0.01, CFI = 1, and SRMR = 0.008. The overall *R* ^2^ for the pathway was 0.16.

The VG level (measured by the VG total score) was high for patients with low TCI-R self-directedness and high TCI-R persistence scores. In addition, TCI-R trait persistence mediated the relationship between age and VG total score: younger subjects had higher TCI-R persistence scores, and a positive association was found between this personality trait and the VG score. TCI-R self-directedness also mediated the relationship between sex and VG total score. Men obtained higher scores on this personality trait, which was negatively associated with VG level.

GD severity (measured by the SOGS-total score) was not associated with VG total score, but it was associated with younger age, low TCI-R self-directedness scores, and high TCI-R persistence scores. Again, as in the case of VG, TCI-R self-directedness mediated the pathway between sex and GD level, and TCI-R persistence mediated the pathway between age and GD level.

## 4. Discussion

The current study assessed the prevalence of VG symptoms in a clinical sample of GD patients and explored the differences between VG groups (VGU versus VGA). Furthermore, we assessed the associations between the severity of VG symptoms and GD symptomatology, general psychopathology and personality traits, and clinical variables and then compared them with patients without VG use (non-VGU).

The main finding of the study was that the prevalence of VGA in a consecutive clinical sample of treatment-seeking GD individuals was 15%. This is in agreement with the literature, which describes an association between the presence of gambling problems and a more frequent use of and involvement in video games [51]. Moreover, our results show that the prevalence of VG problem use or addiction among GD patients is higher than in other similar studies, which ranged from 0.6% to 10%, despite our sample being older [16, 52]. However, the rates obtained in our study are consistent with those described in an adult population [13].

The presence of VG use (VGU and VGA) was associated with specific clinical variables such as younger age, but not with GD symptomatology as measured by means of SOCS or DSM-IV criteria. Previous literature reports suggest that age and gender are strong predictors of problematic or addictive use of video games [13, 20, 51], but not of the severity of the main GD [51, 52].

The second main finding was that both VGU and VGA patients presented higher general psychopathology. This is in agreement with the existing literature [28, 53], which reports an association between a higher number of VG symptoms and depression, anxiety, and social phobia. These emotional disturbances and social problems not only may be consequences of video game addiction [16] but may also be factors that contribute to the persistence of the disorder. Indeed, Kuss [11] describes how the preference for online social relationships, the need for escapism, and use of maladaptive coping strategies to deal with daily stressors become maintaining variables. Similarly, King and Delfabbro [54] consider the problematic use of video games to be associated with attempts to achieve self-esteem or to gain social acceptance.

A third main finding was that patients who made excessive use of VG (both VGU and VGA) presented more dysfunctional personality traits, namely, lower self-directedness and higher persistence. Other studies have also found specific personality traits such as irritability/aggression, impulsivity, neuroticism, loneliness, and introversion to be associated with VGA [52, 55].

The present study has several methodological limitations that need to be taken into account. First, the participants in the sample are only representative of GD patients who seek treatment, and therefore the findings obtained may not apply to all individuals with GD. Since only 7% to 12% of GD individuals seek help for their disorder, a community sample of GD might yield different results. Second, the use of a standardized self-administered questionnaire as the assessment procedure did not allow for an in-depth evaluation of specific axis I and axis II comorbid disorders.

## 5. Conclusions

This study adds to the limited literature on VGA in GD clinical samples and develops a pathway model to describe the associations between VG symptoms, clinical and sociodemographic characteristics, personality traits, and general psychopathology. Based on the findings of the model, we conclude that both VGU and VGA are driven by high levels of persistence and low levels of self-directedness, and that patients tend to be male and of younger age. Intervention strategies that focus on the training of these personality features and systematic screening for potential VGU/VGA are recommended.

## Figures and Tables

**Table 1:** Sociodemographic and clinical characteristics of the GD sample (*N* = 193) and comparisons between groups.

	Total	^ 1^Non-VGU	^ 1^VGU	^ 1^VGA	*P*
	*n* = 193	*n* = 121	*n* = 43	*n* = 29	
Gender; *n* (%)					
Males	167 (86.5%)	103 (85.1%)	39 (90.7%)	25 (86.2%)	0.654
Females	26 (13.5%)	18 (14.9%)	4 (9.3%)	4 (13.8%)	
Age (years); mean (SD)	42.4 (13.4)	45.2 (13.6)	37.3 (12.0)	38.6 (11.1)	0.001
Employed; *n* (%)	99 (51.3%)	61 (50.4%)	23 (53.5%)	15 (51.7%)	0.941
Marital status: single; *n* (%)	64 (33.2%)	37 (30.6%)	16 (37.2%)	11 (37.9%)	0.613
Smoker; *n* (%)	109 (56.5%)	66 (54.5%)	23 (53.5%)	20 (69.0%)	0.336
Use of alcohol; *n* (%)	35 (18.1%)	20 (16.5%)	7 (16.3%)	8 (27.6%)	0.358
Use of substances; *n* (%)	14 (7.3%)	10 (8.3%)	3 (7.0%)	1 (3.4%)	0.666
Age of onset PG problems; mean (SD)	15.7 (10.8)	17.2 (11.5)	11.7 (9.0)	15.4 (9.2)	0.024
Duration of PG; mean (SD)	5.94 (7.0)	5.87 (6.8)	5.03 (7.5)	7.58 (7.0)	0.370
Main gambling; *n* (%)					
Slot machines	123 (63.7%)	77 (63.6%)	26 (60.5%)	20 (69.0%)	0.762
Bingo	12 (6.2%)	11 (9.1%)	1 (2.3%)	0 (0%)	
Lotteries	13 (6.7%)	11 (9.1%)	1 (2.3%)	1 (3.4%)	
Casino	8 (4.1%)	5 (4.1%)	3 (7.0%)	0 (0%)	
Other	37 (19.2%)	17 (14.0%)	12 (27.9%)	8 (27.6%)	

SD: standard deviation. ^1^Non-VGU (non-video game users) (total VDT score of 0); VGU: video game users (total VDT score between 1 and 19); VGA: video game addicts (total VDT score of 20 or higher). Chi-square test for categorical outcomes and ANOVA for quantitative outcomes.

**Table 2:** Comparison for SOGS scores and DSM-IV criteria.

	^ 1^Non-VGU	^ 1^VGU	^ 1^VGA	Group	LT	QT	Effect size		
	*n* = 121	*n* = 43	*n* = 29	*P*	*P*	*P*	^ 2^Cohen's |*d*|		
(1a) Card games; %	27.35%	33.33%	52.17%	0.064	**0.022**	0.547	0.13	**0.52∗**	0.39
(1b) Horse racing; %	3.42%	0%	0%	0.338	0.998	0.999	0.27	0.27	—
(1c) Sports events; %	3.42%	7.69%	0%	0.287	0.998	0.998	0.19	0.27	0.41
(1d) Lottery/scratchcards; %	84.62%	84.62%	91.30%	0.697	0.409	0.585	0.00	0.21	0.21
(1e) Casino; %	24.79%	41.03%	26.09%	0.145	0.895	0.087	0.35	0.03	0.32
(1f) Bingo; %	51.28%	46.15%	47.83%	0.841	0.762	0.729	0.10	0.07	0.03
(1g) Stock market; %	5.13%	5.13%	0%	0.539	0.998	0.998	0.00	0.33	0.33
(1h) Slot machines; %	81.20%	89.74%	100%	**0.045**	0.998	0.998	0.24	**0.68∗**	0.48
(1i) Other forms of gambling; %	15.38%	5.26%	31.82%	**0.022**	0.072	**0.032**	0.34	0.39	0.73
(2) Amount of money spent: ≥300 euros; %	53.85%	66.67%	52.17%	0.341	0.883	0.162	0.26	0.03	0.30
(3) Family history of gambling; %	22.22%	15.38%	34.78%	0.208	0.204	0.132	0.18	0.28	0.46
(4) Going back to win back lost money; %	91.38%	89.74%	100%	0.307	0.998	0.998	0.06	0.43	0.48
(5) Claiming to be winning when losing; %	40.87%	43.59%	56.52%	0.385	0.171	0.607	0.06	0.32	0.26
(6) Problem recognition; %	97.44%	97.44%	100%	0.740	0.998	0.998	0.00	0.23	0.23
(7) Gambling more than planned; %	91.38%	92.31%	100%	0.347	0.998	0.998	0.03	0.43	0.41
(8) Being criticized; %	66.09%	74.36%	82.61%	0.230	0.127	0.919	0.18	0.39	0.20
(9) Feeling guilty; %	95.73%	100%	100%	0.256	0.998	0.999	0.30	0.30	—
(10) Inability to stop gambling; %	92.24%	92.31%	100%	0.385	0.998	0.998	0.00	0.41	0.41
(11) Hiding signs of gambling; %	69.83%	74.36%	78.26%	0.662	0.417	0.992	0.10	0.19	0.09
(12) Arguments with family/friends; %	78.63%	79.49%	91.30%	0.369	0.175	0.394	0.02	0.36	0.34
(13) Arguments and fights; %	74.53%	71.43%	73.91%	0.937	0.951	0.757	0.07	0.01	0.06
(14) Borrowing money and failing to return it; %	46.15%	41.03%	52.17%	0.691	0.598	0.408	0.10	0.12	0.22
(15) Skipping work due to gambling; %	43.59%	46.15%	65.22%	0.163	0.062	0.396	0.05	0.44	0.39
(16a) Taking money from home; %	77.78%	77.27%	93.33%	0.375	0.199	0.330	0.01	0.45	0.47
(16b) Taking money from partner; %	59.52%	60.00%	83.33%	0.300	0.143	0.379	0.01	**0.55∗**	0.54
(16c) Taking money from family; %	77.08%	86.96%	87.50%	0.541	0.514	0.704	0.26	0.28	0.02
(16d) Borrowing from banks; %	81.48%	77.78%	83.33%	0.918	0.880	0.679	0.09	0.05	0.14
(16e) Using credit cards; %	77.78%	91.67%	84.62%	0.323	0.589	0.279	0.39	0.18	0.22
(16f) Borrowing from money lenders; %	30.30%	45.45%	0%	0.239	0.999	0.999	0.32	**0.93∗**	**1.29∗**
(16g) Money from sale of shares or other bank assets; %	7.69%	14.29%	25.00%	0.553	0.311	1.000	0.21	0.48	0.27
(16h) Money from property sales; %	41.67%	50.00%	20.00%	0.552	0.369	0.348	0.17	0.48	**0.66∗**
(16i) Money from making out false checks; %	8.00%	0%	0%	0.654	0.999	1.000	0.42	0.42	—

SOGS: total score; mean (SD)	9.66 (3.2)	10.1 (3.6)	11.2 (2.4)	0.117	**0.043**	0.670	0.13	**0.54∗**	0.36

DSM1. Preoccupations with gambling; %	73.95%	80.95%	86.96%	0.318	0.191	0.964	0.17	0.33	0.16
DSM2. Need to bet more money; %	62.18%	42.86%	86.96%	**0.002**	**0.031**	**0.001**	0.39	**0.59∗**	**1.04∗**
DSM3. Unsuccessful efforts to control; %	90.76%	95.24%	100%	0.229	0.998	0.998	0.18	0.45	0.32
DSM4. Restless, irritable when not gambling; %	59.66%	71.43%	73.91%	0.227	0.203	0.639	0.25	0.31	0.06
DSM5. Gambling to escape from problems; %	73.95%	64.29%	82.61%	0.255	0.382	0.102	0.21	0.21	0.42
DSM6. Gambling again after losing money; %	73.95%	80.95%	95.65%	0.062	**0.050**	0.342	0.17	**0.63∗**	0.47
DSM7. Lying to family members or others; %	90.76%	95.24%	86.96%	0.494	0.578	0.260	0.18	0.12	0.29
DSM8. Committing illegal acts; %	27.73%	28.57%	30.43%	0.965	0.792	0.955	0.02	0.06	0.04
DSM9. Losing a significant relationship, job,…; %	78.99%	80.95%	91.30%	0.387	0.184	0.478	0.05	0.35	0.30
DSM10. Relying on others to provide money; %	70.59%	83.33%	69.57%	0.250	0.922	0.116	0.31	0.02	0.33

DSM. Total criteria; mean (SD)	7.03 (2.3)	7.24 (1.9)	8.04 (1.5)	0.114	**0.038**	0.471	0.10	**0.52∗**	0.47

SD: standard deviation. ^1^Non-VGU (non-video game users) (total VDT score of 0); VGU: video game users (total VDT score between 1 and 19); VGA: video game addicts (total VDT score of 20 or higher). LT: linear trend; QT: quadratic trend. ^2^Cohen's |*d*| for the comparisons: non-VGU versus VGU; non-VGU versus VGA; VGU versus VGA. ∗Bold: moderate (|*d* | >0.50) to high (|*d* | >0.80) effect size.

**Table 3:** Comparison for clinical outcomes.

	^ 1^Non-VGU		^ 1^VGU		^ 1^VGA		ANOVA					
	*n* = 121		*n* = 43		*n* = 29		Group	Trends		Effect size		
	Mean	SD	Mean	SD	Mean	SD	P	LT	QT	^ 2^Cohen's |*d*|		
SCL-90: somatization	1.13	0.87	0.95	0.91	1.69	1.09	**0.003**	**0.030**	**0.008**	0.20	**0.57∗**	**0.74∗**
SCL-90: obsessive/compulsive	1.20	0.88	1.12	0.78	1.96	0.93	**<0.001**	**0.001**	**0.005**	0.10	**0.84∗**	**0.98∗**
SCL-90: interpersonal sensitivity	1.18	0.94	1.05	0.87	1.89	0.94	**<0.001**	**0.004**	**0.006**	0.14	**0.76∗**	**0.93∗**
SCL-90: depression	1.66	0.96	1.56	0.92	2.21	0.93	**0.010**	**0.026**	**0.036**	0.11	**0.58∗**	**0.70∗**
SCL-90: anxiety	1.15	0.86	1.03	0.83	1.74	0.97	**0.002**	**0.011**	**0.013**	0.14	**0.64∗**	**0.79∗**
SCL-90: hostility	1.00	0.88	0.80	0.76	1.67	1.08	**<0.001**	**0.007**	**0.002**	0.24	**0.68∗**	**0.93∗**
SCL-90: phobic anxiety	0.53	0.70	0.43	0.76	0.99	0.96	**0.007**	**0.027**	**0.023**	0.14	**0.55∗**	**0.65∗**
SCL-90: paranoia	1.02	0.84	0.98	0.78	1.77	0.96	**<0.001**	**<0.001**	**0.010**	0.05	**0.83∗**	**0.90∗**
SCL-90: psychoticism	1.06	0.80	0.89	0.75	1.58	1.03	**0.002**	**0.027**	**0.007**	0.22	**0.56∗**	**0.77∗**
SCL-90: GSI score	1.18	0.76	1.06	0.72	1.79	0.87	**<0.001**	**0.004**	**0.003**	0.16	**0.75∗**	**0.91∗**
SCL-90: PST score	49.43	21.25	48.90	21.45	65.07	18.88	**<0.001**	**0.002**	**0.035**	0.02	**0.78∗**	**0.80∗**
SCL-90: PSDI score	1.99	0.64	1.80	0.56	2.34	0.64	**0.002**	**0.007**	**0.002**	0.32	**0.55∗**	**0.90∗**

TCI-R: novelty seeking	108.36	12.21	108.51	12.92	110.22	12.39	0.778	0.529	0.744	0.01	0.15	0.14
TCI-R: harm avoidance	104.03	15.93	98.90	20.80	106.52	16.46	0.157	0.996	0.054	0.28	0.15	0.41
TCI-R: reward dependence	98.92	13.70	101.62	10.23	98.11	13.97	0.466	0.883	0.220	0.22	0.06	0.29
TCI-R: persistence	103.54	23.10	114.79	21.65	112.89	23.13	**0.012**	**0.010**	0.135	**0.50∗**	0.40	0.08
TCI-R: self-directedness	131.27	20.93	132.77	21.02	117.56	18.56	**0.005**	**0.012**	**0.037**	0.07	**0.69∗**	**0.77∗**
TCI-R: cooperativeness	132.95	16.40	132.69	15.48	125.78	15.23	0.107	0.068	0.282	0.02	0.45	0.45
TCI-R: self- transcendence	62.79	15.35	60.69	12.82	66.89	17.14	0.261	0.410	0.156	0.15	0.25	0.41

VG: total score	0.00	0.00	6.77	4.60	44.24	22.60	**<0.001**	**<0.001**	**<0.001**	**2.08∗**	**2.77∗**	**2.30∗**

SD: standard deviation. LT: linear trend; QT: quadratic trend.

^1^Non-VGU (non-video game users) (total VDT score of 0); VGU: video game users (total VDT score between 1 and 19); VGA: video game addicts (total VDT score of 20 or higher).

^2^Cohen's |*d*| for the comparisons: non-VGU versus VGU; non-VGU versus VGA; VGU versus VGA. ∗Bold: moderate (|*d* | >0.50) to high (|*d* | >0.80) effect size.

**Table 4:** Partial correlations, adjusted for participants' sex and age, between VG total score and clinical outcomes.

SCL-90: somatization	**0.248**
SCL-90: obsessive/compulsive	**0.295**
SCL-90: interpersonal sensitivity	**0.291**
SCL-90: depression	**0.221**
SCL-90: anxiety	**0.258**
SCL-90: hostility	**0.274**
SCL-90: phobic anxiety	**0.270**
SCL-90: paranoid ideation	**0.319**
SCL-90: psychoticism	**0.245**
SCL-90: GSI score	**0.297**
SCL-90: PST score	**0.266**
SCL-90: PSDI score	**0.227**

TCI-R: novelty seeking	0.085
TCI-R: harm avoidance	0.089
TCI-R: reward dependence	−0.055
TCI-R: persistence	0.091
TCI-R: self-directedness	**−0.195**
TCI-R: cooperativeness	−0.104
TCI-R: self-transcendence	0.118

Bold: significant correlation (.05 level).

**Table 5:** Predictive models for the video game questionnaire scores through step-wise regression.

Linear regression for outcome:	Predictors	B	β	P	95% CI (*B*)	
VG-total scale score	TCI-R: self-directedness	−0.172	−0.199	0.007	−0.296;	−0.048

Logistic regression for outcome:	Predictors	*B*	OR	*P*	95% CI (OR)	

VGU	Age (years)	−0.041	0.960	0.003	0.934;	0.986
	TCI-R: Persistence	0.016	1.016	0.033	1.001;	1.030

VGA	TCI-R: self-directedness	−0.036	0.965	0.002	0.943;	0.987

VGU: video game users (total VDT score between 1 and 19); VGA: video game addicts (total VDT score of 20 or higher).

**Table 6:** Structural equation model.

	Standard coefficient	SE	*Z*	*P* > |*Z*|	95% CI for the coefficient	
TCI-R persistence						
Sex (male)	0.1068688	0.0756534	1.41	0.0158	−0.0414092;	0.2551468
Age (years)	−0.2368213	0.0732199	−3.23	0.001	−0.3803297;	−0.0933129
_Constant	5.10091	0.4282882	11.91	<0.001	4.261481;	5.94034
TCI-R self-directedness						
Sex (male)	0.1483216	0.0773418	1.92	0.050	0.0032656;	0.2999087
Age (years)	0.0519053	0.0784299	0.66	0.508	−0.1018144;	0.2056251
_Constant	5.614348	0.5221618	10.75	<0.001	4.590929;	6.637766
VG-total score						
TCI-R persistence	0.1575215	0.0763331	2.06	0.039	0.0079114;	0.3071316
TCI-R self-directedness	−0.2375955	0.0728751	−3.26	0.001	−0.3804281;	−0.0947629
Sex (male)	0.030922	0.0768931	0.40	0.688	−0.1197858;	0.1816298
Age (years)	−0.0700488	0.0776069	−0.90	0.367	−0.2221556;	0.082058
_Constant	1.293971	0.6827771	1.90	0.058	−0.0442471;	2.63219
SOGS-total score						
TCI-R persistence	0.1196749	0.0733896	1.63	0.103	−0.0241661;	0.2635159
TCI-R self-directedness	−0.3278795	0.0694936	−4.72	0.000	−0.4640846;	−0.1916745
TDV_TOTAL	0.0034844	0.0732836	0.05	0.962	−0.1401489;	0.1471176
Sex (male)	0.0359377	0.0726224	0.49	0.621	−0.1063995;	0.1782749
Age (years)	−0.2235272	0.0713199	−3.13	0.002	−0.3633117;	−0.0837427
_Constant	5.114255	0.634818	8.06	<0.001	3.870035;	6.358476

	*χ* ^2^	*P*	RMSEA	CFI	SRMR	

Model level goodness-of-fit	0.291	0.589	0.01	1.00	0.008	

Equation level goodness-of-fit	Fitted	Variance	Residual	*R* ^2^	mc	mc^2^

TCI-R persistence	535.6957	41.93446	493.7613	0.0782804	0.2797863	0.0782804
TCI-R self-directedness	438.6029	9.393018	429.2099	0.0214158	0.1463413	0.0214158
VG-total score	302.4843	28.20133	274.283	0.0932324	0.3053398	0.0932324
SOGS-total score	10.77644	2.064861	8.711581	0.1916088	0.4377314	0.1916088

mc = correlation between dependent variable and its prediction.

mc^2^ = Bentler-Raykov squared multiple correlation coefficient.
